# Supplementary material for: The Human Soluble NKG2D Ligand Differentially Impacts Tumorigenicity and Progression in Temporal and Model-Dependent Modes
Source: Biomedicines. 2024 Jan 16;12(1):196. doi: 10.3390/biomedicines12010196 (PMC10812945; doi:10.3390/biomedicines12010196)
Supplement: Supplementary file 1 [file biomedicines-12-00196-s001.zip › biomedicines-2736238-supplementary.pptx]

## Slide 1
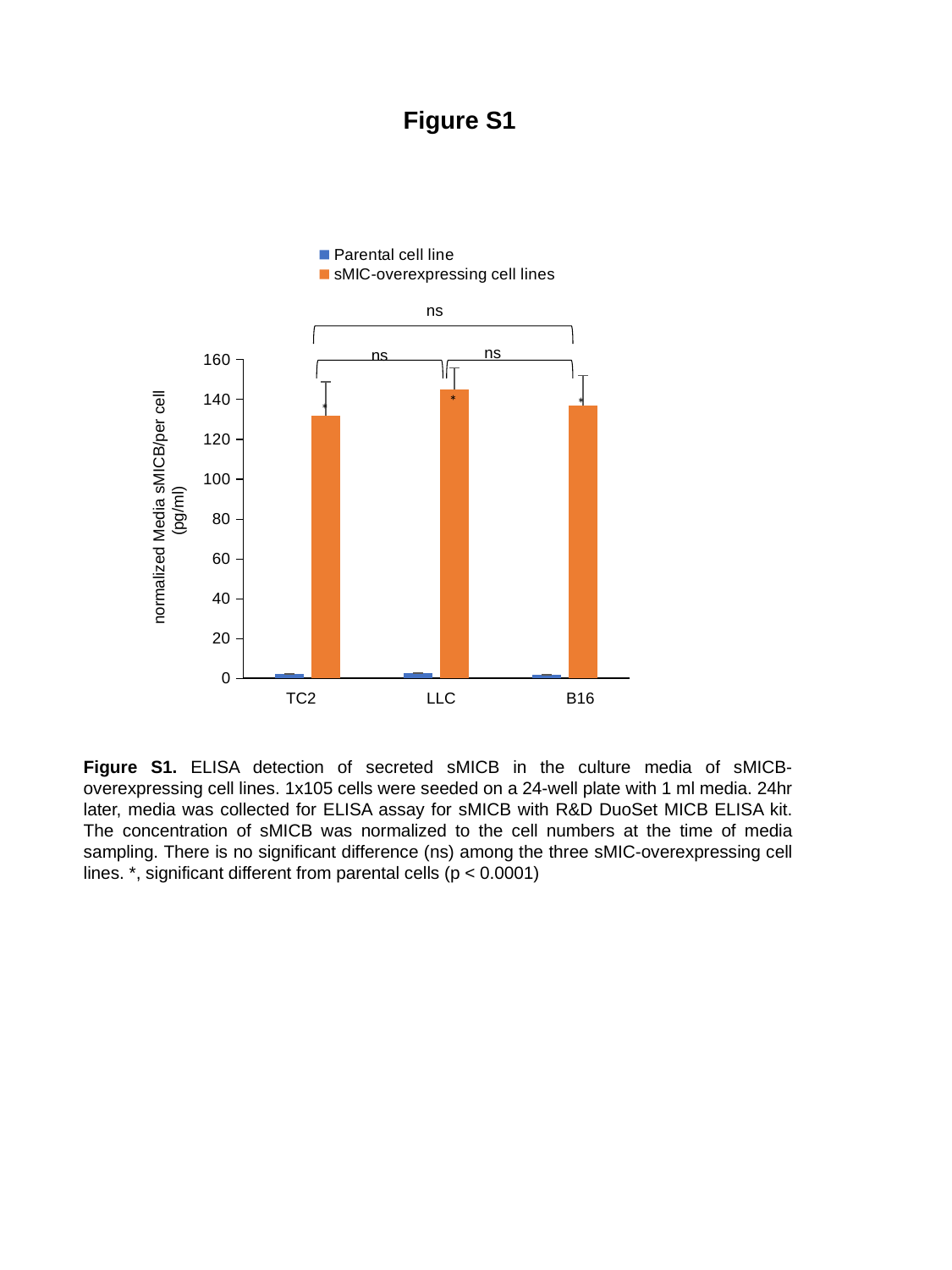

Figure S1
### Chart
| Category | | |
|---|---|---|ns
ns
ns
*
*
*
normalized Media sMICB/per cell
 (pg/ml)
TC2
LLC
B16
Figure S1. ELISA detection of secreted sMICB in the culture media of sMICB-overexpressing cell lines. 1x10^5^ cells were seeded on a 24-well plate with 1 ml media. 24hr later, media was collected for ELISA assay for sMICB with R&D DuoSet MICB ELISA kit. The concentration of sMICB was normalized to the cell numbers at the time of media sampling. There is no significant difference (ns) among the three sMIC-overexpressing cell lines. *, significant different from parental cells (p < 0.0001)

## Slide 2
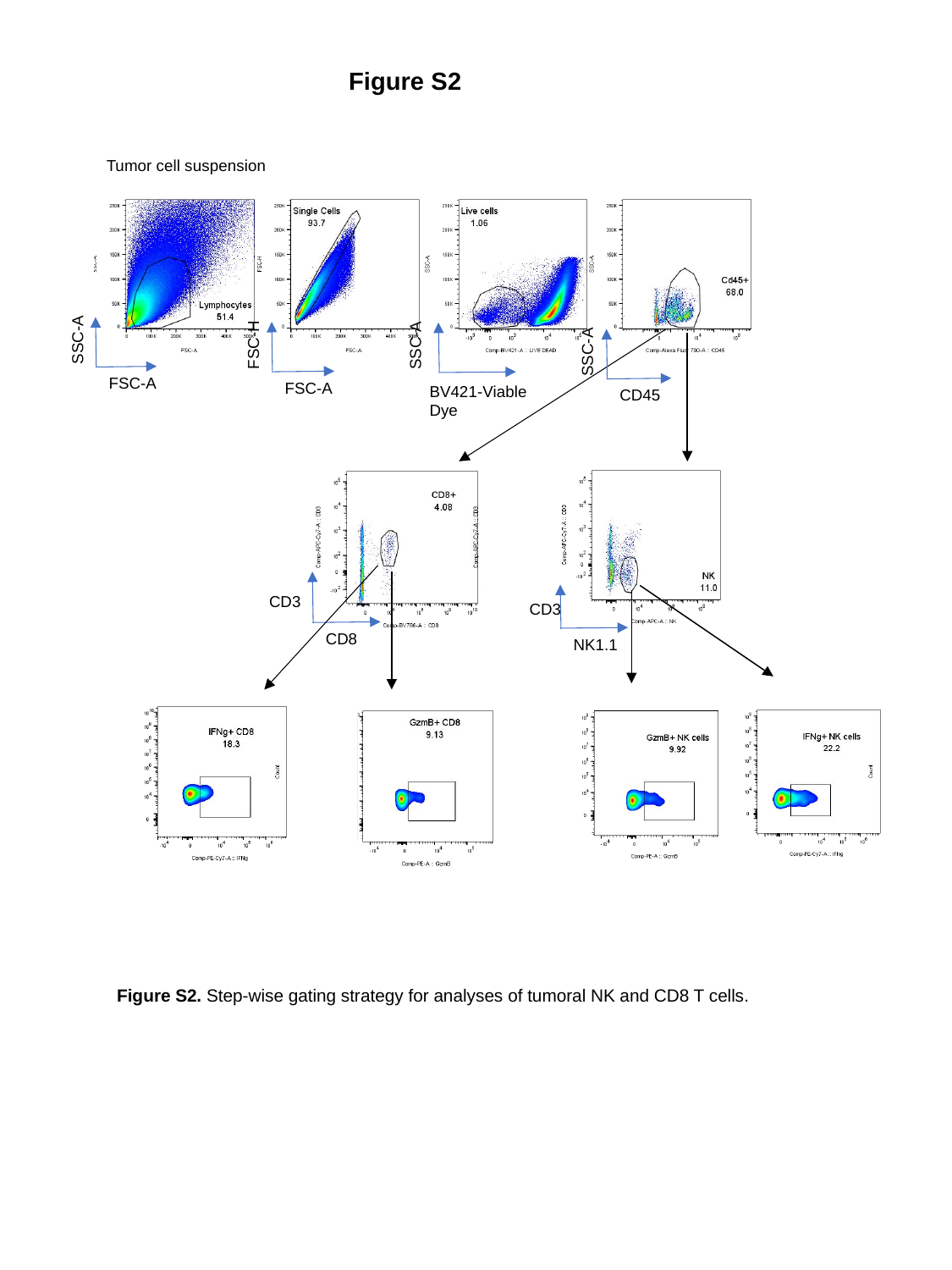

Figure S2
Tumor cell suspension
SSC-A
FSC-A
FSC-H
FSC-A
SSC-A
BV421-Viable Dye
SSC-A
CD45
CD3
CD8
CD3
NK1.1
Figure S2. Step-wise gating strategy for analyses of tumoral NK and CD8 T cells.

## Slide 3
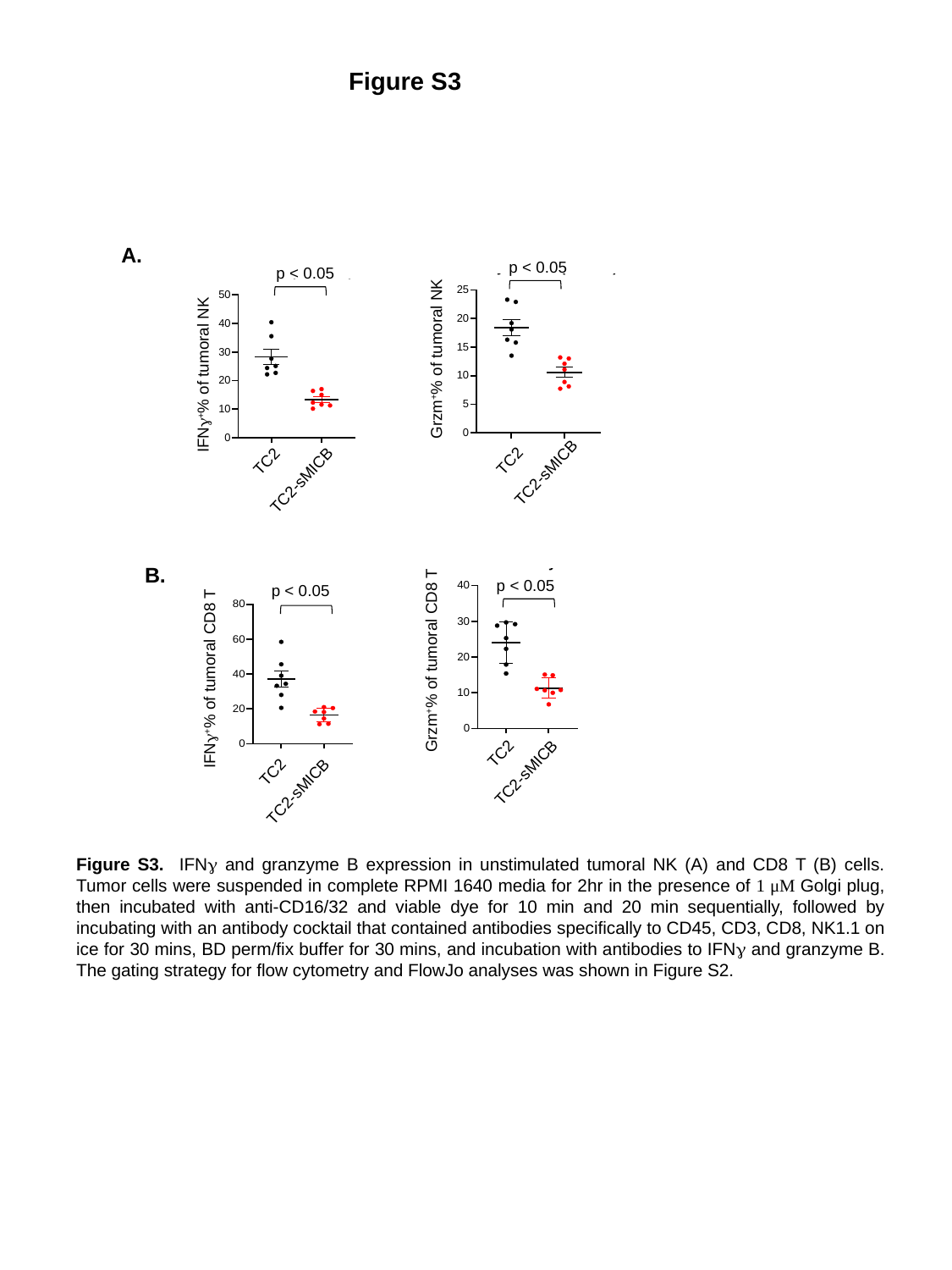

Figure S3
A.
p < 0.05
p < 0.05
Grzm+% of tumoral NK
IFNg+% of tumoral NK
TC2
TC2
TC2-sMICB
TC2-sMICB
p < 0.05
p < 0.05
Grzm+% of tumoral CD8 T
IFNg+% of tumoral CD8 T
TC2
TC2
TC2-sMICB
TC2-sMICB
B.
Figure S3. IFNg and granzyme B expression in unstimulated tumoral NK (A) and CD8 T (B) cells. Tumor cells were suspended in complete RPMI 1640 media for 2hr in the presence of 1 μM Golgi plug, then incubated with anti-CD16/32 and viable dye for 10 min and 20 min sequentially, followed by incubating with an antibody cocktail that contained antibodies specifically to CD45, CD3, CD8, NK1.1 on ice for 30 mins, BD perm/fix buffer for 30 mins, and incubation with antibodies to IFNg and granzyme B. The gating strategy for flow cytometry and FlowJo analyses was shown in Figure S2.

## Slide 4
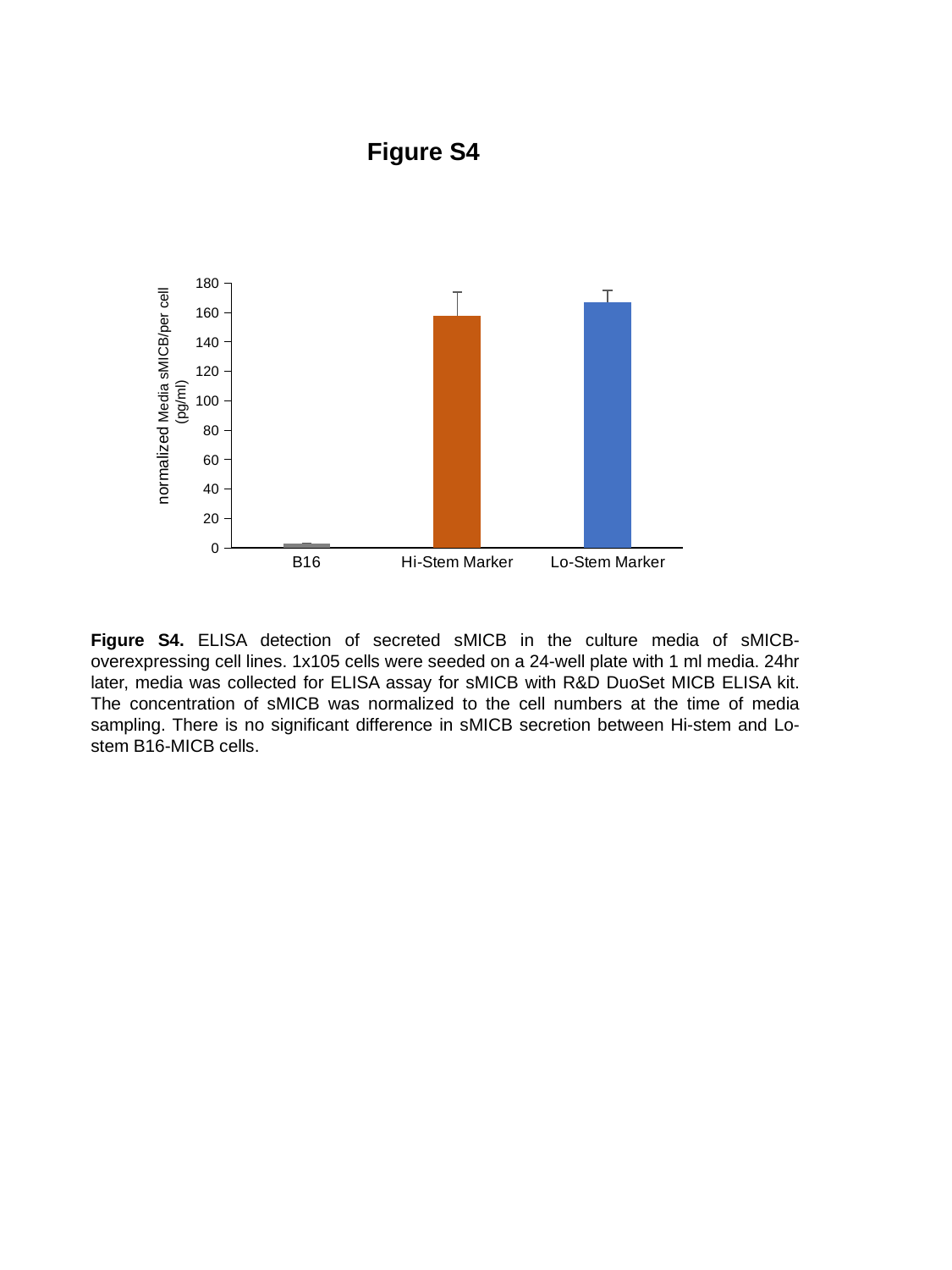

Figure S4
### Chart
| Category | |
|---|---|
| B16 | 2.9 |
| Hi-Stem Marker | 158.0 |
| Lo-Stem Marker | 167.0 |normalized Media sMICB/per cell
 (pg/ml)
Figure S4. ELISA detection of secreted sMICB in the culture media of sMICB-overexpressing cell lines. 1x10^5^ cells were seeded on a 24-well plate with 1 ml media. 24hr later, media was collected for ELISA assay for sMICB with R&D DuoSet MICB ELISA kit. The concentration of sMICB was normalized to the cell numbers at the time of media sampling. There is no significant difference in sMICB secretion between Hi-stem and Lo-stem B16-MICB cells.
